# One-anastomosis gastric bypass (OAGB) versus Roux-en-Y gastric bypass (RYGB) as revisional procedures after failed laparoscopic sleeve gastrectomy (LSG): systematic review and meta-analysis of comparative studies

**DOI:** 10.1007/s00423-023-03175-x

**Published:** 2023-11-18

**Authors:** Antonio Vitiello, Giovanna Berardi, Roberto Peltrini, Pietro Calabrese, Vincenzo Pilone

**Affiliations:** 1 Advanced Biomedical Sciences Department, University of Naples Federico II, Via S. Pansini 5, 80131 Naples, Italy; 2 Public Health Department, University of Naples Federico II, Via S. Pansini 5, 80131 Naples, Italy

**Keywords:** Revisional surgery, One-anastomosis gastric bypass, Roux-en-Y gastric bypass, Sleeve gastrectomy, GERD

## Abstract

**Introduction:**

The aim of this study was to compare weight loss and gastroesophageal reflux disease (GERD) remission after one-anastomosis gastric bypass (OAGB) versus Roux-en-Y gastric bypass (RYGB) as revisional procedures after laparoscopic sleeve gastrectomy (LSG).

**Methods:**

In PubMed, Embase, and Cochrane Library, a search was performed using the terms “Roux-en-Y gastric bypass versus one anastomosis gastric bypass,” “revisional surgery,” and “sleeve gastrectomy.” Only original articles in English language comparing OAGB and RYGB were included. No temporal interval was set. The primary outcome measure was weight loss (%TWL). The secondary endpoints were leak, bleeding, marginal ulcer, and GERD. PRISMA flowchart was used. Differences in continuous and dichotomous outcome variables were expressed as mean difference (MD) and risk difference (RD) with 95% CI, respectively. Heterogeneity was assessed by using *I*^2^ statistic.

**Results:**

Six retrospective comparative articles were included in the present meta-analysis. Weight loss analysis showed a MD = 5.70 (95% CI 4.84–6.57) in favor of the OAGB procedure with a statistical significance (*p* = 0.00001) and no significant statistical heterogeneity (*I*^2^ = 0.00%). There was no significant RD for leak, bleeding, or marginal ulcer after the two revisional procedures. After conversion to OAGB, remission from GERD was 68.6% (81/118), and it was 80.6% (150/186) after conversion to RYGB with a RD = 0.10 (95% CI −0.04, 0.24), no statistical significance (*p* = 0.19), and high heterogeneity (*I*^2^ = 96%). De novo GERD was 6.3% (16/255) after conversional OAGB, and it was 0.5% (1/180) after conversion to RYGB with a RD = −0.23 (95% CI −0.57, 0.11), no statistical significance (*p *= 0.16), and high heterogeneity (*I*^2^ = 92%).

**Supplementary Information:**

The online version contains supplementary material available at 10.1007/s00423-023-03175-x.

## Introduction

Laparoscopic sleeve gastrectomy (LSG) is currently the most performed bariatric procedure worldwide [1]. Despite this popularity, LSG was reported to be associated with weight regain and gastroesophageal reflux disease (GERD) in the long-term with a revision rate up to 36% [2]. Some articles have also described intestinal metaplasia (Barrett’s disease) after LSG due to the chronic exposure of the lower esophagus to reflux [3, 4]. Roux-en-Y gastric bypass (RYGB) and one-anastomosis gastric bypass (OAGB) are, respectively, the second and the third most performed interventions, and they have both been suggested as good options for failed LSG [2–5]. Specifically, RYGB is considered an efficient treatment for GERD post-LSG [6], while OAGB may provide better results in terms of further weight loss [7].

The aim of this study was to analyze and compare weight loss and GERD remission after OAGB versus RYGB as revisional procedures after LSG.

## Methods

Preferred Reporting Items for Systematic reviews and Meta-Analyses (PRISMA) guidelines were followed [8].

### Literature search

In PubMed, Embase, and Cochrane Library, a search was performed using the terms “Roux-en-Y gastric bypass versus one anastomosis gastric bypass,” “revisional surgery,” and “sleeve gastrectomy.” In addition, the reference lists of all retrieved articles were manually reviewed. According to Problem/Population, Intervention, Comparison, and Outcome (PICO) framework, study selection criteria were exactly defined. Only original articles in English language comparing OAGB and RYGB were included. No temporal interval was set. The primary outcome measure was weight loss. The secondary endpoints were leak, bleeding, GERD remission, and de novo reflux. The last search was performed in December 2022.

### Studies selection

Two independent authors analyzed each article and performed data extraction independently. Duplicate studies were removed. In case of disagreement, further investigation was conducted by an additional author.

### Statistical analysis

DataRev software (Cochrane) version 5.4.1 (the Cochrane Collaboration 2011, the Nordic Cochrane Centre, Copenhagen) was used to perform a random-effect meta-analysis with Mantel–Haenszel calculation because of the observational nature of most studies included in this analysis.

Differences in continuous and dichotomous outcome variables were expressed as mean difference and risk difference (RD) with 95% CI, respectively. Heterogeneity was assessed by using *I*^2^ statistic, which describes the percentage of total variation across studies that is due to heterogeneity rather than chance. Usually, values of the *I*^2^ statistic < 25% are indicative of low heterogeneity, those ranging between 25 and 75% of moderate heterogeneity, and those > 75% of high heterogeneity. *I*^2^ < 40% was considered as non-important heterogeneity. A *p* < 0.05 was considered statistically significant. Publication bias was assessed through visual inspection of funnel plots.

### Quality assessment

The Newcastle–Ottawa Quality Assessment Scale (NOS) [9] was used as an assessment tool to evaluate case–control studies. The scale’s range varies from 0 to 9 stars, and studies with a score equal to or higher than 5 were considered to have an adequate methodological quality to be included.

## Results

**Fig. 1:**
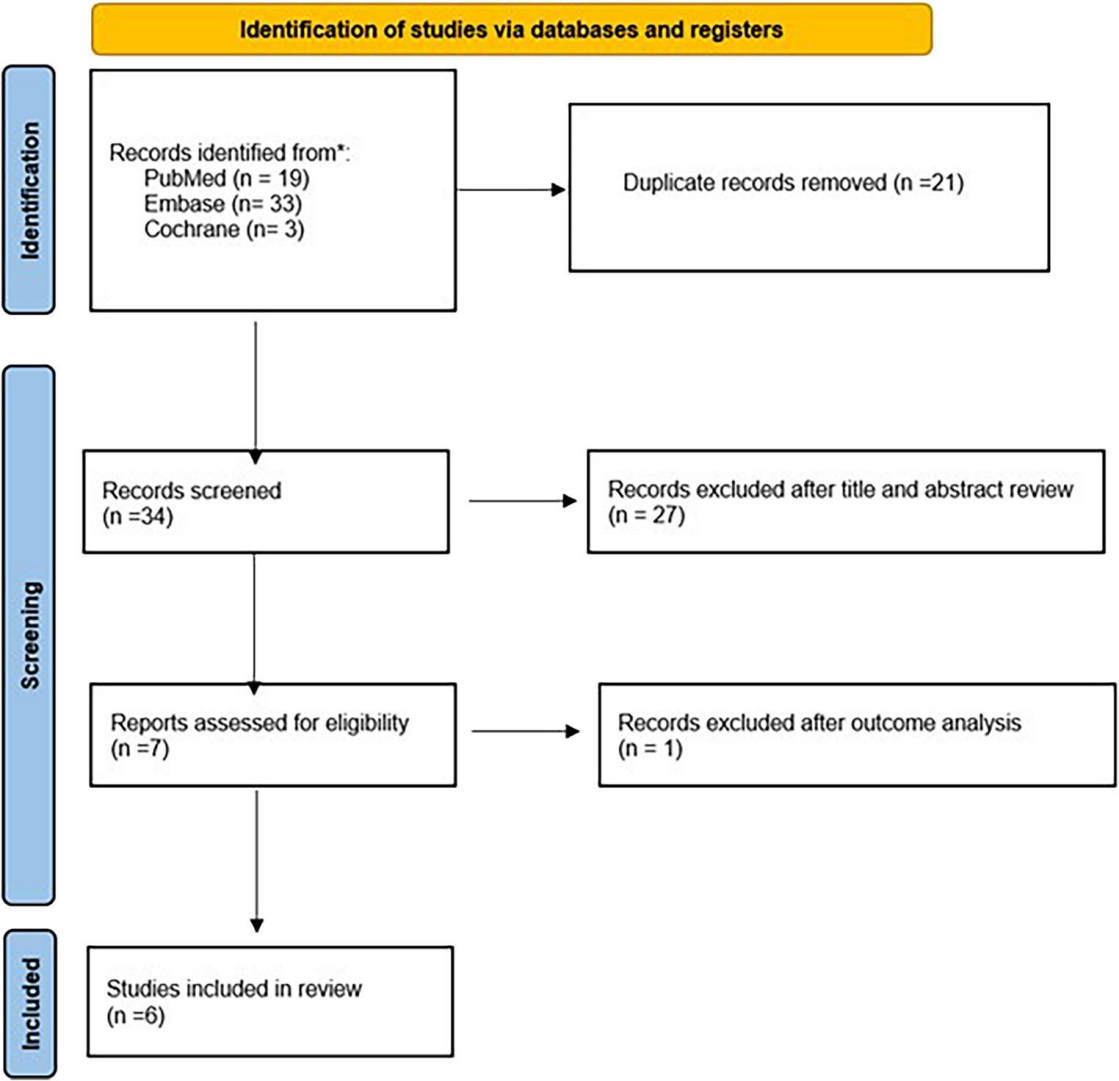
PRISMA flowchart

The literature search found 55 articles. After removal of 21 duplicates, other 27 articles were excluded because they were not comparing RYGB and OAGB as revisional procedures. Seven [10–15] papers were considered eligible, but one [16] was excluded due to incomplete report of the outcome measures. PRISMA flow chart for the study selection is shown in Fig. 1. Eventually, 5 retrospective articles and 1 randomized controlled trial were included in our meta-analysis (Table 1). In total, 739 patients were included, of which 373 (50.5%) underwent OAGB and 366 (49.5%) underwent RYGB. The sample size of these studies ranged from 55 to 263 patients. The primary outcome measure was reported both as percentage of total weight loss (%TWL) and percentage of excess weight loss (%EWL) or excess BMI loss percent (%EBMIL) with a follow-up ranging from 12 to 60 months; assessment with NOS showed high-quality methodology for all the considered papers (Table 2).

**Table 1:** Included studies and baseline characteristics

Study (year)	Primary surgery	Revisional surgery	Patient (*n*)	Age (years)	Male (*n*)	BMI at conversion (kg/m^2^)	Max follow-up time (months)	BMI at follow-up (kg/m^2^)
Chiappeta (2019)	LSG	OAGB	34	46.76 ± 11.48	11	45.7 ± 8	12	36.6 ± 6.3
		RYGB	21	46.14 ± 10.8	2	36.6 ± 6.9	12	33.5 ± 5.6
Rayman (2021)	LSG	OAGB	144	42.4 ± 10.5	37	41.6 ± 5.7	25.5	31.8 ± 5.3
		RYGB	119	44.3 ± 11.8	35	39.6 ± 5.0	35	33.3 ± 5.0
Felsenreich (2022)	LSG	OAGB	13	-	-	45.0 ± 7.3	15	31.4 ± 8.1
		RYGB	45	-	-	38.6 ± 8.6	15	30.3 ± 8.5
Rheinwalt (2022)	LSG	OAGB	55	42 ± 1.3	33	45.5 ± 1.0	24	35
		RYGB	68	46 ± 1.2		39.3 ± 1.0 kg	24	31
Wilczyński (2022)	LSG	OAGB	47	45.02 ± 10.71	13	40.44 ± 5.8	60	-
		RYGB	33	41.24 ± 8.906	6	38.70 ± 6.84	60	-
Hany (2022)	LSG	OAGB	80	42.6 ± 7.1	11	45.1 ± 8.3	24	27.4 ± 3.1
		RYGB	80	43.4 ± 7.5	11	44.9 ± 6.6	24	27.8 ± 2.2

**Table 2:** Outcomes of the included studies

Study (year)	Revisional surgery	Operative time (min)	Sample (*n*)	Leaks (*n*, %)	Bleeding (*n*, %)	Marginal ulcer (*n*, %)	EWL%	TWL%	T2DM resolution (%)	HTN resolution (%)	GERD on follow-up (%)	NOS
Chiappeta (2019)	OAGB	79 ± 36	34	0 (0%)	0 (0%)	0 (0%)	29 ± 13	15.8 ± 7.8	100%	66.7%	11.8%	9
	RYGB	98 ± 24	21	0 (0%)	0 (0%)	1 (4.8%)	22 ± 18	10.3 ± 7.6	60%	0%	4.8%	
Rayman (2021)	OAGB	-	144	2 (1.4%)	2(1.4%)	0 (0%)	58.7	32 ± 9	-	-	17.4%	9
	RYGB	-	119	1 (1.7%)	3 (2.5%)	0 (0%)	44.2	27 ± 9	-	-	7.6%	
Felsenreich (2022)	OAGB	-	13	0 (0%)	0 (0%)	0 (0%)	80.3 ± 23.7	39.5 ± 11.5	-	-	28.9%	9
	RYGB	-	45	0 (0%)	0 (0%)	0 (0%)	79.8 ± 34.1	37.7 ± 14.6	-	-	53.8%	
Rheinwalt (2022)	OAGB	168 ± 7.2	55	2 (3.6%)	0 (0%)	0 (0%)	50	24 ± 2.6	92%	92%	13.34%	8
	RYGB	201 ± 6.8	68	4 (5.9%)	2 (2.9%)	0 (0%)	40	18 ± 3.0	100%	89%	11.1%	
Wilczyński (2022)	OAGB	-	47	0 (0%)	1 (2.12%)	3 (6.4.%)	84.04 ± 18.81	21.81 ± 12.48	97.3%	27.3%	28.6%	8
	RYGB	-	33	0 (0%)	1 (3%)	4 (12.1%)	72.95 ± 20.3	18.39 ± 11.85	33.3%	30%	60%	
Hany (2022)	OAGB	85.6 ± 18.6	80	0 (0%)	1 (1.25%)	0 (0%)	-*	-*	75%	68%	-	8
	RYGB	104.9 ± 13.7	80	0 (0%)	1 (1.25%)	2 (2.5%)	-*	-*	71%	75%	-	

*NOS* Newcastle–Ottawa Scale. *T2SM* type 2 diabetes. *HTN* hypertension. ^*^Hany et al. reported weight loss as %EBMIL

**Fig. 2:**
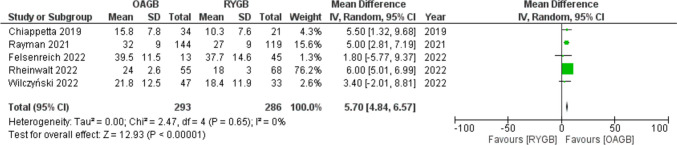
Forest plot for percentage of total weight loss

Weight loss was reported using different parameters, but percentage of total weight loss (%TWL) was used in five studies showing a MD = 5.70 (95% CI 4.84–6.57) in favor of the OAGB procedure with a statistical significance (*p* < 0.001) and no significant statistical heterogeneity (*I*^2^ = 0%) (Fig. 2).

Overall leak rate after conversion to OAGB was 1% (4/373), and it was 1.6% (6/366) after revision to RYGB.

**Fig. 3:**
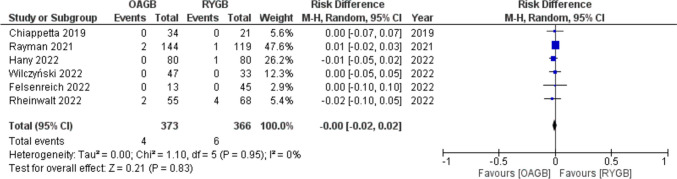
Forest plot for leak

Meta-analysis showed a RD =  − 0.00 (95% CI − 0.02–0.02) with no statistical significance (*p* = 0.83) and no significant statistical heterogeneity (*I*^2^ = 0.00%) (Fig. 3).

**Fig. 4:**
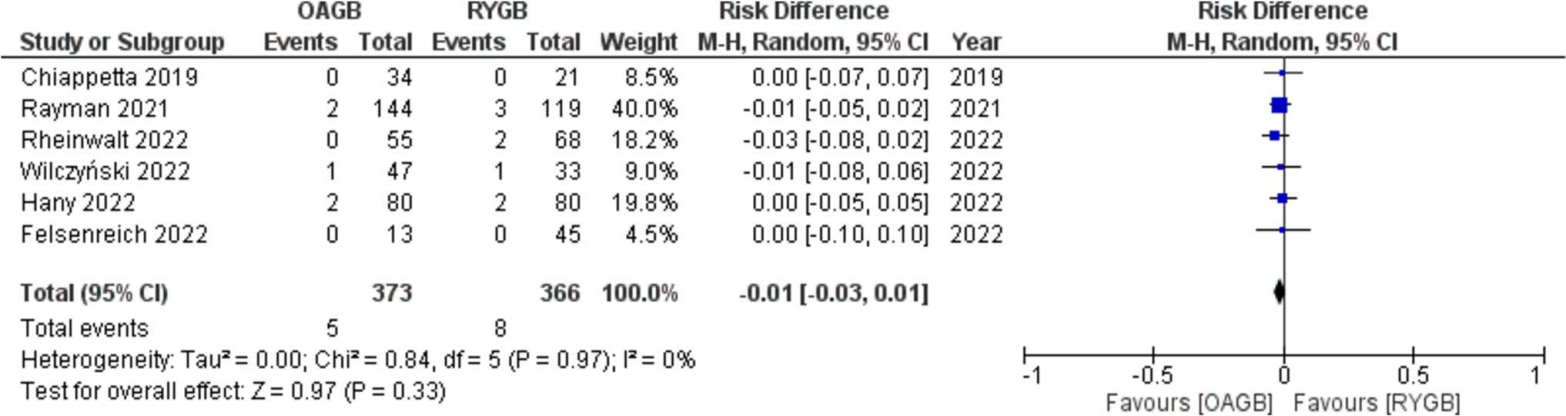
Forest plot for bleeding

Total bleedings after revisional OAGB and RYGB were 1.3% (5/373) and 2.2% (8/366), respectively, with a RD =  − 0.01 (95% CI − 0.03, 0.01) with no statistical significance (*p* = 0.33) and no significant statistical heterogeneity (*I*^2^ = 0.00%) (Fig. 4).

**Fig. 5:**
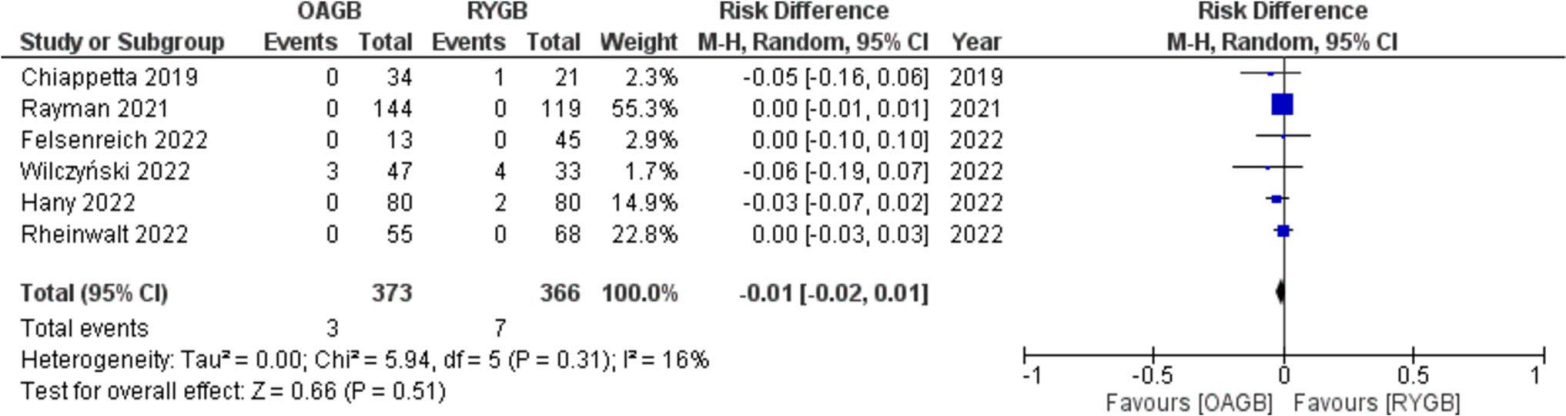
Forest plot for marginal ulcer

Total percentage of marginal ulcers after conversion to OAGB was 0.8% (3/373), and it was 1.9% (7/366) after revision to RYGB. Meta-analysis showed a RD =  − 0.01 (95% CI − 0.02, 0.01) with no statistical significance (*p* = 0.51) and low heterogeneity (*I*^2^ = 16%) (Fig. 5).

**Fig. 6:**
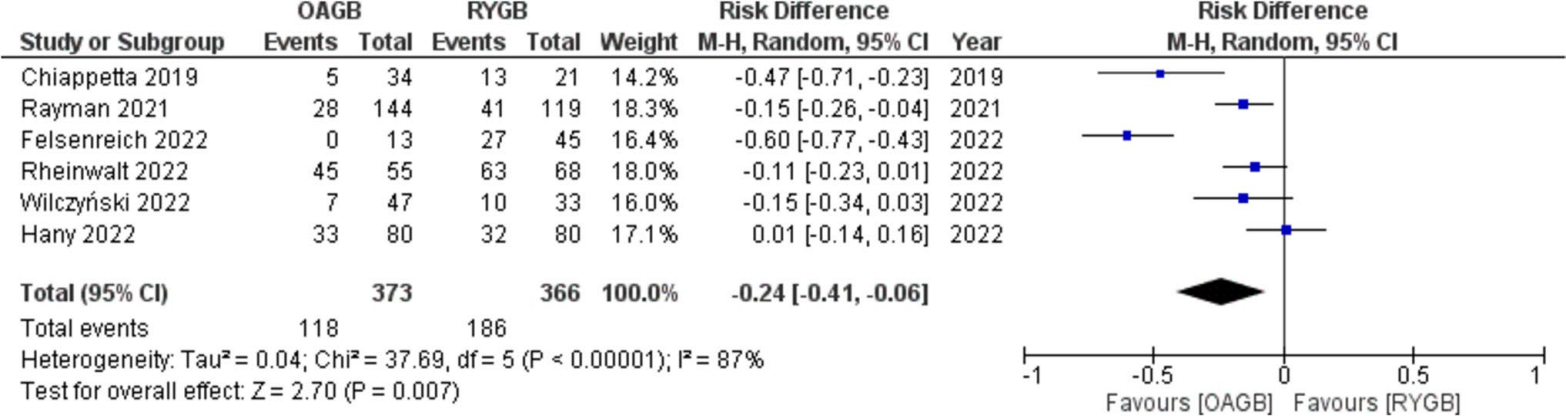
Forest plot for GERD as indication for conversion

GERD was the indication for conversion for 31.6% (118/373) of patients before OAGB and for 50.8% (186/366) before RYGB. Meta-analysis of rate of preconversional GERD showed a RD =  − 0.24 (95% CI − 0.41, − 0.06) with statistical significance (*p* = 0.007) and high heterogeneity (*I*^2^ = 87%) (Fig. 6).

**Fig. 7:**
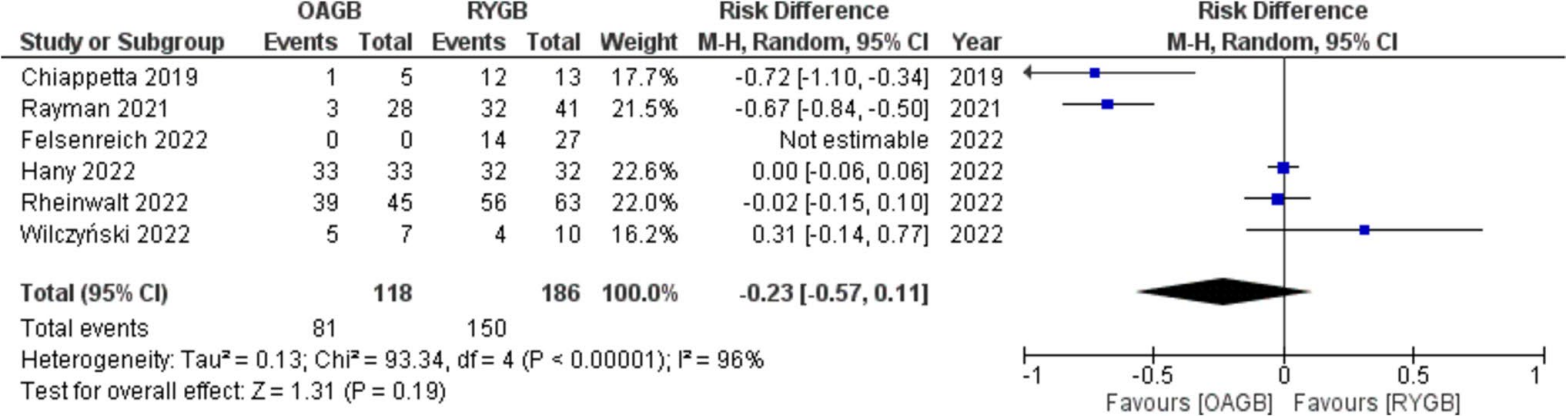
Forest plot for GERD after conversion

After conversion to OAGB remission from GERD was 68.6% (81/118), and it was 80.6% (150/186) after conversion to RYGB with a RD = 0.10 (95% CI − 0.04, 0.24) with no statistical significance (*p* = 0.19) and high heterogeneity (*I*^2^ = 96%) (Fig. 7).

**Fig. 8:**
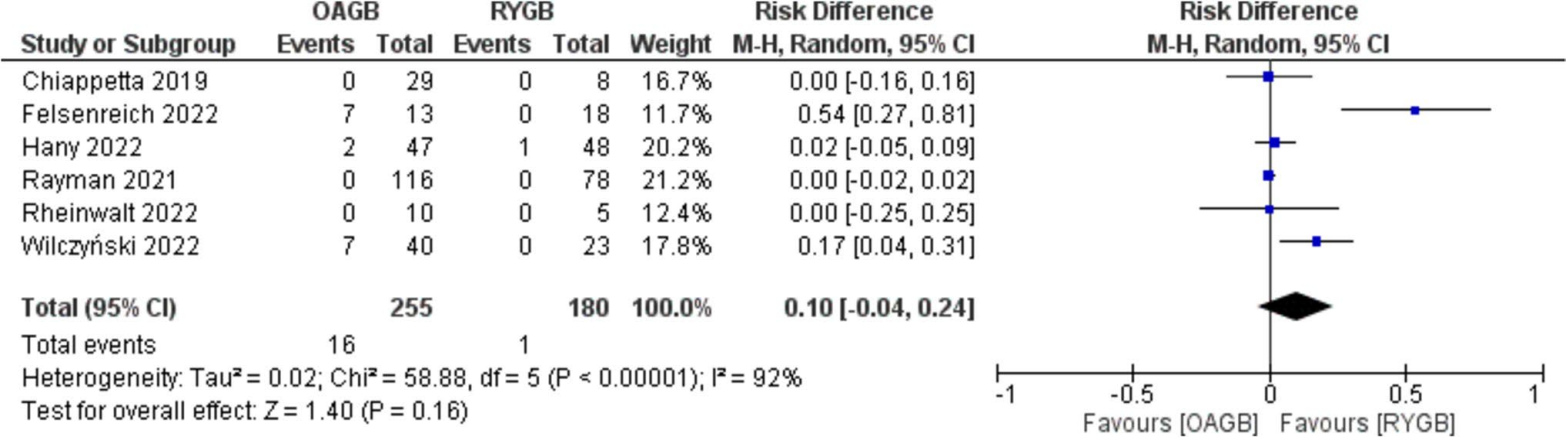
Forest plot for de novo GERD after conversion

De novo GERD was 6.3% (16/255) after conversional OAGB, and it was 0.5% (1/180) after conversion to RYGB with a RD =  − 0.23 (95% CI − 0.57, 0.11) with no statistical significance (*p* = 0.16) and high heterogeneity (*I*^2^ = 92%) (Fig. 8).

Funnel plots inspection did not show significant bias (Supplement materials 1–6).

## Discussion

LSG was initially introduced by Marceau [17] and Gagner [18] proposed as a first step of a staged procedure in patients with BMI > 60 kg/m [2]. Since postoperative outcomes demonstrated low morbidity and satisfactory weight loss, LSG became a stand-alone bariatric intervention [19]. Short-term studies (1–3 years) reported an excess weight loss (%EWL) comparable to the values of the RYGB [20]. Mid-term reports (5–7 years) have shown less successful results, with a certain percentage of weight regain [21, 22]; the SM-BOSS [23] study showed that excess BMI loss peaked at 2 years after SG (74.7%) but decreased by the end of the fifth year to 61.1%.

Recently, long-term studies have demonstrated a worrisome rate of conversion and GERD [24], especially in individuals with BMI > 50 kg/m^2^ [25]. Sporadic cases of vitamin deficiency after LSG have been also published [26].

A recent systematic review showed a rate of de novo GERD of 20% [27] after LSG, while a meta-analysis found that the increase of postoperative GERD was 19%, and de novo reflux occurred in 23% [28] of patients.

Despite several meta-analyses have investigated the role of OAGB and RYGB as revisional procedures after failed restrictive surgery [6, 7, 29], there is a lack of comparative studies on the role of this interventions specifically after failed LSG. Chiappetta et al. [10] first reported their single-center analysis of 55 patients showing that OAGB after failed SG was a quicker procedure with less perioperative complications. On the contrary, Rayman [12] reported that conversion of LSG to OAGB, compared to RYGB, resulted in increased weight loss with a higher rate of GERD and potential nutritional deficiencies. Instead, Felsenreich et al. [11] have recently concluded that with regard to the fact that OAGB has a low potential to cure patients from GERD symptoms after SG, RYGB is probably the best option for patients post-LSG reflux. Rheinwalt [13] also found comparable results with significantly shorter operation times for OAGB. After a follow-up of 5 years, Wilczyński[14] reported a significant remission of T2DM after OAGB when compared to RYGB after LSG. Hany et al. [15] have performed the only available controlled trial demonstrating that after 2 years, both revisional RYGB and OAGB have comparable metabolic outcomes.

Our analysis has demonstrated a low-to-moderate heterogeneity among these studies with a high-quality methodology. Weight loss as TWL%, EWL%, or %EBMIL and rates of early complications (leak, bleeding) were reported in all the papers. Regardless of the used parameter, the mean weight loss after one-anastomosis gastric bypass was higher than after RYGB in all but one of the included articles; thus, the present meta-analysis confirmed the inferiority of RYGB in terms of weight loss. Only Rheinwalt [13] found that the two interventions induced comparable weight loss probably for the long biliopancreatic limb of the RYGB in this study.

Low rates of early complications (leak, bleeding) found in all the collected papers demonstrated the feasibility and safety of revisional surgery after LSG.

Regarding long-term complications, some authors have reported a higher occurrence of marginal ulcer (MU) after revisional surgery [30] especially due to the risk of retained gastric antrum syndrome (RGA) after conversion to gastric bypass [31, 32]. Conversely, in this systematic review, after a follow-up ranging from 12 to 60 months, the rate of MU was 1% both for RYGB and OAGB.

As expected, we found that a higher rate of patients with GERD after LSG was converted to RYGB rather than to OAGB, but remission from GERD was satisfactory and comparable after the two procedures. Even if de novo GERD occurred more frequently after revisional OAGB, new-onset reflux and Barrett’s disease were reported after both revisional interventions.

### Strength and limitations

Although a meta-analysis [33] was recently published, the present includes two more papers (6 instead of 4) and focuses not only on weight loss but also on the safety (early complications) and on GERD symptoms after revision. The main limitation is that GERD was assessed through different diagnostic methods with a lack of information on severity of GERD, presence and size of eventual hiatal hernia, and degree of esophagitis. Moreover, several revisional procedures were performed together with a concomitant hiatoplasty, which may have influenced the results on reflux. This is particularly interesting for the treatment of patients with severe obesity suffering from GERD and/or hiatal hernia (HH). Even if from 22 to 37% of class three obesity patients have a hiatal hernia (HH) [34], these defects are preoperatively underdiagnosed or not repaired intraoperatively. Conversely, studies with long-term results have demonstrated that SG plus hiatal hernia repair (HHR) induces symptoms relief up to 60% of patients [35]. Considering that GERD itself is a major issue before and after SG, HHR should be considered mandatory for those with severe obesity and GERD undergoing sleeve gastrectomy. Eventually, we must acknowledge that weight loss is mostly influenced by the length of the biliopancreatic limb; therefore, future studies comparing OAGB and RYGB after LSG should take into account the bypassed lengths of small bowel.

## Conclusion

Conversion from LSG to RYGB or OAGB is feasible and safe with a low rate of postoperative complications.

Despite weight loss is satisfactory after both procedures, OAGB provides better results. Remission from GERD is higher after RYGB but without statistical significance.

Without knowing the applied bypass length in most of the analyzed studies, OAGB might be a better option for failed LSG, while RYGB still should be preferred in case of severe GERD.

### Supplementary Information

Below is the link to the electronic supplementary material.

Supplementary file1 (PNG 3 KB)

Supplementary file2 (PNG 3 KB)

Supplementary file3 (PNG 3 KB)

Supplementary file4 (PNG 3 KB)

Supplementary file5 (PNG 3 KB)

Supplementary file6 (PNG 3 KB)

## Data Availability

All data are available online since this is a systematic review of published studies.
