# Supplementary figures and images for: One-anastomosis gastric bypass (OAGB) versus Roux-en-Y gastric bypass (RYGB) as revisional procedures after failed laparoscopic sleeve gastrectomy (LSG): systematic review and meta-analysis of comparative studies
Source: Langenbecks Arch Surg. 2023 Nov 18;408(1):440. doi: 10.1007/s00423-023-03175-x (PMC10657303; doi:10.1007/s00423-023-03175-x)

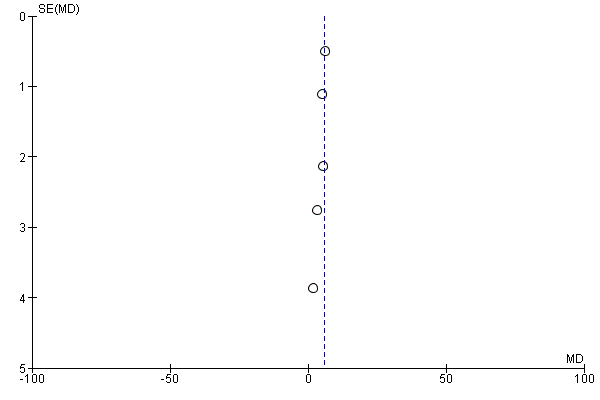

Supplement: Supplementary file 1 — Supplementary file1 (PNG 3 KB) [file 423_2023_3175_MOESM1_ESM.png]

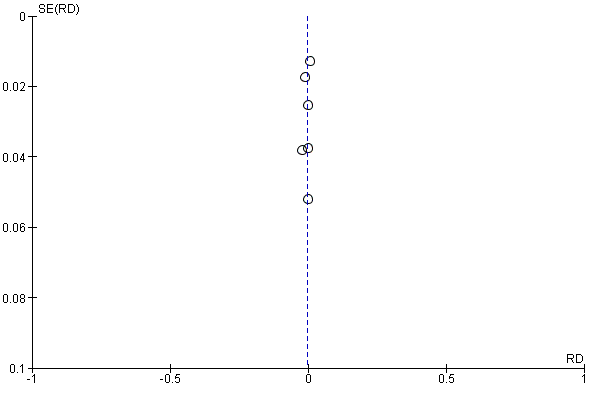

Supplement: Supplementary file 2 — Supplementary file2 (PNG 3 KB) [file 423_2023_3175_MOESM2_ESM.png]

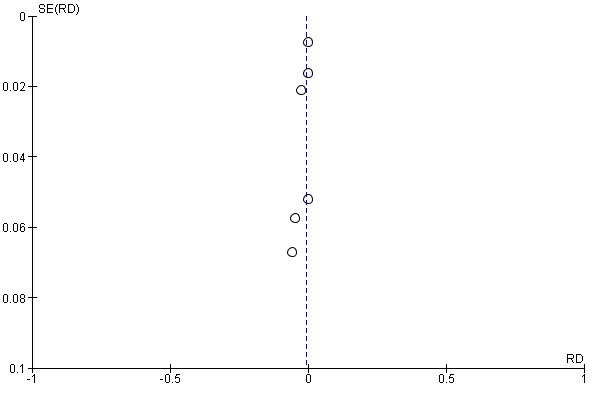

Supplement: Supplementary file 3 — Supplementary file3 (PNG 3 KB) [file 423_2023_3175_MOESM3_ESM.png]

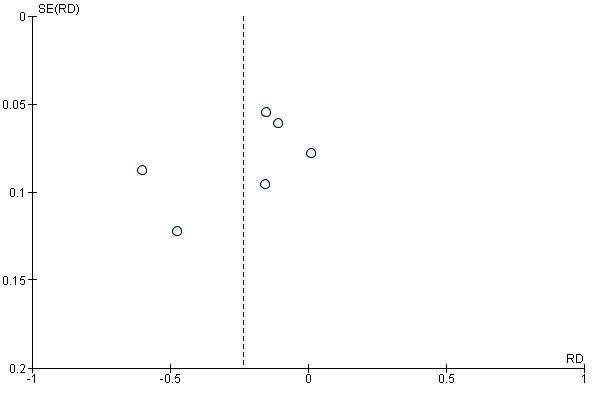

Supplement: Supplementary file 4 — Supplementary file4 (PNG 3 KB) [file 423_2023_3175_MOESM4_ESM.png]

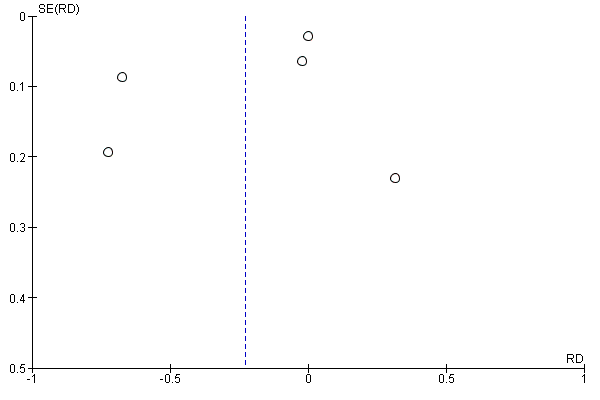

Supplement: Supplementary file 5 — Supplementary file5 (PNG 3 KB) [file 423_2023_3175_MOESM5_ESM.png]

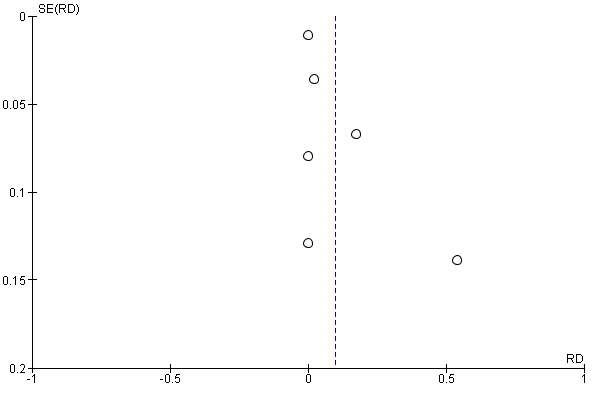

Supplement: Supplementary file 6 — Supplementary file6 (PNG 3 KB) [file 423_2023_3175_MOESM6_ESM.png]
